# Feasibility of amino acid profiling in long-term stored formalin-fixed paraffin-embedded colorectal neoplasia tissue

**DOI:** 10.1007/s11306-025-02301-8

**Published:** 2025-07-27

**Authors:** Roza C. M. Opperman, Puck E. Bruchner, Sofie Bosch, Tim G. J. de Meij, Evelien Dekker, Nanne K. H. de Boer, Eduard A. Struys

**Affiliations:** 1 Department of Gastroenterology and Hepatology, Amsterdam UMC, Vrije Universiteit Amsterdam, 1081 HV Amsterdam, The Netherlands; 2 Amsterdam Gastroenterology Endocrinology Metabolism (AGEM) Research Institute, 1081 HV Amsterdam, The Netherlands; 3 Cancer Center Amsterdam, research program, 1081 HV Amsterdam, The Netherlands; 4 Department of Pediatric Gastroenterology, Emma Children’s Hospital, Amsterdam UMC, Vrije Universiteit Amsterdam, 1081 HV Amsterdam, The Netherlands; 5 Department of Pediatric Gastroenterology, Emma Children’s Hospital, Amsterdam UMC, Academic Medical Centre, 1105 AZ Amsterdam, The Netherlands; 6 Department of Gastroenterology and Hepatology, Amsterdam UMC, University of Amsterdam, 1081 HV Amsterdam, The Netherlands; 7 Department of Laboratory Medicine, Amsterdam University Medical Centre, 1105 AZ Amsterdam, The Netherlands

**Keywords:** Colorectal cancer, Colorectal adenoma, Amino acids, Liquid-chromatography tandem-mass spectrometry, Formalin-fixed paraffin embedded

## Abstract

**Introduction:**

Metabolic processes play a role in cancer development, with faecal amino acids emerging as potential biomarkers for colorectal neoplasia. While fresh frozen tissue is preferred for metabolomic analysis, formalin-fixed paraffin-embedded (FFPE) tissue is more widely available.

**Objectives:**

We aimed to evaluate amino acid profiles in FFPE tissue across different stages of the adenoma-carcinoma sequence.

**Method:**

A panel of 20 amino acids was measured using liquid chromatography–tandem mass spectrometry.

**Results:**

Fourteen amino acids were detected, with proline elevated in colorectal carcinoma versus advanced (FC 2.33, *p* = 0.04) and non-advanced adenomas (FC 2.42, *p* = 0.02).

**Conclusion:**

Despite analytical challenges, amino acid profiling in FFPE tissue is feasible.

**Supplementary Information:**

The online version contains supplementary material available at 10.1007/s11306-025-02301-8.

## Introduction

With growing evidence that metabolic processes contribute to cancer development and progression, the study of measuring and quantifying disease-associated metabolites is rapidly expanding (Danzi et al., 2023). Previous studies have highlighted the potential of faecal amino acids as non-invasive biomarkers for colorectal neoplasia detection, and analysing their levels in tissue may offer valuable insights into their involvement in the adenoma-carcinoma sequence (Bosch et al., 2022a, b). Amino acids may be involved in various cancer-related pathways, including those related to cell proliferation, immune modulation (e.g., via tryptophan metabolism), and activation of growth-regulating signalling pathways such as mTOR (Zhu et al., 2025). In contrast to genetic or protein-based biomarkers, amino acids may reflect real-time metabolic shifts and microenvironmental influences, making them potentially more sensitive in early disease detection (Siminska & Koba, 2016). However, metabolites, including amino acids, are typically analyzed in fresh frozen tissue, which is preserved at -80 ℃ immediately after collection. This analytical approach is mainly conducted in research settings and this technique is therefore limited to prospective sample collection.

In clinical settings, diagnostic tissue samples are most often preserved using formalin fixation followed by paraffin embedding (FFPE). After processing, FFPE tissue can be stored at room temperature for prolonged periods of time, without the need for specialized equipment such as -80 ℃ freezers. This makes FFPE a practical and cost-effective method for long-term tissue archiving and a suitable source for retrospective metabolomics studies. Pathologists can precisely identify dysplastic regions within FFPE polyps and carcinomas, ensuring that metabolite analysis is focused on the relevant tissue areas. Polyps may be only a few millimeters in diameter, presenting analytical challenges in measuring metabolites in such small sample sizes.

Currently, no standardized protocol exists for analyzing metabolites in FFPE tissue. Literature describes various methods involving high-temperature exposure during deparaffinization of tissue, which may, however, lead to protein degradation and alteration of amino acid composition (Chardin et al., 2023; Isaiah et al., 2024; Yuan et al., 2012). Several studies have explored metabolite detection in FFPE tissue, mostly using untargeted approaches that offer broad but less specific insights. Whether individual amino acids can be reliably quantified using a targeted method remains unclear. In this explorative study, we aim to investigate the feasibility to measure amino acids in minimal amounts of FFPE tissue using a targeted LC-MS/MS method and to evaluate whether there are differences in amino acid concentrations between the different stages of the adenoma-carcinoma sequence.

## Method

### Patient selection

For this study, FFPE tissue samples derived from participants were selected from a previous cohort study (Bosch et al., 2022a). Inclusion criteria required that these participants had undergone a colonoscopy at the Amsterdam University Medical Centre between February 2016 and November 2019, during which a non-advanced adenoma (nAA; 0.5–0.9 cm, no high-grade dysplasia or villous histology at histopathology), an advanced adenoma (AA; ≥ 1 cm in diameter, with or without villous histology or high-grade dysplasia), or colorectal carcinoma (CRC) was removed. Diagnosis was based on histopathological examination of the FFPE tissue.

### Sample preparation

A dedicated gastrointestinal pathologist performed histopathologic evaluation of the tissue slices to identify the dysplastic area in each tissue block. To minimize contamination, an initial 10 μm tissue slice was removed and discarded from each block. For tissue blocks containing sufficient dysplastic tissue, biopsies of 2 × 5 mm were extracted. In cases where dysplastic tissue was insufficient for a biopsy, four slices of 10 μm thickness were taken per block instead. After deparaffinization, the dysplastic tissue was scraped from the slides for further processing. To account for potential background signals, a blank slide (without tissue) underwent the same processing steps and served as a control. In total, 26 samples were processed using the biopsy approach and 3 samples using the scraping approach, depending on tissue availability. All subsequent steps were performed using a standardized protocol to ensure consistency across samples, regardless of the sampling method. Deparaffinization and purification of the FFPE tissue specimen were performed as described elsewhere (Buszewska-Forajta et al., 2019). Briefly, 1 ml of xylene was added to the specimen, followed by vortexing for 1 min and centrifugation for 5 min (18,620 g and 4 ℃). The xylene was then removed, and this procedure was repeated three times. To purify the sample, 0.7 ml of 96% ethanol was added, vortexed for 1 min, and centrifuged again for 5 min (18,620 g and 4 ℃). The ethanol was discarded, and 500 µl of distilled water was added to the specimen. To ensure disruption of cells, a Beadmill homogenisation procedure was employed. In brief, aqueous mixture was transferred to a 2 ml Bead Mill vial containing ceramic beads (Fisherbrand, USA), and placed in the Bead Mill homogeniser (Fisherbrand Bead Mill 24, USA) for 5 s with a speed of 4.85 m/s. After subsequent centrifugation, the resulting mixture was used for targeted amino acid analysis.

### Targeted amino acid analysis

In total, 20 amino acids were targeted, based on our previous studies, including alanine, α-aminoadipic acid, citrulline, glutamic acid, glutamine, glycine, histidine, isoleucine, leucine, lysine, methionine, ornithine, phenylalanine, proline, serine, taurine, threonine, tryptophan, tyrosine, and valine (Bosch et al., 2022a, b). Amino acid analysis was conducted using stable-isotope dilution LC-MS/MS. In total, 30 µL sample was vortex mixed with 10 µl of 10 times diluted internal standard mixture (ChromSystems). Next, to obtain FMOC-derivatives of the studied amino acids, 100 µl borate buffer (65 mM, pH 11) and 100 µl FMOC-Cl in acetone (15 mg/10 ml) were added, followed by mixing. A Vanquish-UPLC system (Thermo Scientific) coupled to TSQ Quantiva tandem mass spectrometer (Thermo Scientific) was used to detect and quantify the amino acids. The separation of the amino acids was carried out using a Water BEH C18 UPLC column (1.7 μm particle size). The column is directly coupled to a Quantiva MS (Thermo Scientific) equipped with an electrospray ionization source. 30µL of a 10 times diluted amino acid panel calibrator (Chromsystems), taken into preparation as described for the processed FFPE extract, served as quantitative reference.

### Statistical analysis

Preferably, amino acid concentrations are expressed per mg protein. However, our attempts to reliably measure these ultra-low protein levels in the final obtained aqueous homogenates, failed. Therefore, we normalized the data by expressing each amino acid concentration as a percentage of the total amino acid content within the same tissue sample. This within-sample normalization accounts for variability in tissue input across samples. All statistical analyses were conducted using R software (version 4.2.1), for data visualization the ggplot 2 package was used. Extreme outliers, defined as values more than three times the interquartile range below the first quartile or above the third quartile, were excluded from subsequent analyses. Differences in median were analysed using the Kruskal-Wallis test, with pairwise Wilcoxon rank-sum test as post hoc analysis. Categorical variables were analysed using the Fisher’s exact test. To explore multivariable patterns, Principal Component Analysis (PCA) was performed on amino acid concentrations, followed by MANOVA on the first two principal components. A P-value ≤ 0.05 were considered significant. Multiple testing correction was not employed to avoid type 2 errors. Potential confounding by sample characteristics was evaluated using Spearman correlation for continuous variables and Mann-Whitney U tests for binary variables.

## Results and discussion

In total, we included 12 non-advanced adenomas, 12 advanced adenomas and 5 colorectal adenocarcinomas (Table 1). Two adenocarcinoma tissue blocks, were excluded from further analysis. Both consisted of dysplastic tissue obtained via scraping, as the blocks did not contain sufficient material for biopsy. The signals detected by LC-MS/MS for these samples were comparable to those of the blank control, indicating insufficient tissue-derived signal for reliable quantification. Samples were stored for a median of 8 years and 10 months [7y9m– 9y2m] at room temperature. From the 20 targeted amino acids, we were able to detect 14 unique amino acids. The amino acids that were targeted but not detected in any of the samples were: α-aminoadipic acid, leucine, tyrosine, tryptophan, isoleucine and histidine.

**Table 1:** Characteristics of participants and lesions

	CRC	Advanced adenoma	Non advanced adenoma	*p*-value
	(*n* = 3)	(*n* = 12)	(*n* = 12)	
Age (median [IQR])	67.0 [63.5–68.0]	65.0 [64.8–67.8]	68.5 [66.8–71.5]	0.37
Sex (n [%])				
Male	3 [100]	12 [100]	6 [50.0]	0.01
Female	0 [0.0]	0 [0.0]	6 [50.0]	
BMI (median [IQR])^a^	26.0 [25.1–26.7]	25.2 [23.2–25.8]	27.3 [26.0-28.4]	0.14
Storage time, years.months (median [IQR])	8.10 [8.6–8.10]	9.2 [9.0-9.2]	7.10 [7.1–8.9]	0.03
Adenoma histologic type (n [%])				
Tubular adenoma	NA	6 [50.0]	12 [100]	
Tubulovillous adenoma	NA	6 [50.0]	0 [0.0]	
Adenoma dysplasia grade (n [%])				
Low	NA	12 [100]	12 [100]	
High	NA	0 [0]	0 [0]	
Location (n [%])				0.60
Distal (descending colon-rectum)	3 [100]	9 [75.0]	7 [58.3]	
Proximal (cecum-splenic flexure)	0 [0]	3 [25.0]	5 [41.7]	
Median lesions size, cm [IQR]	1.2 [0.6–1.5]	1.25 [1.0-1.5]	0.6 [0.55–0.65]	

^a^Three participants having non-advanced adenomas had missing data on weight and length

Our finding that histidine could not be detected in FFPE tissue aligns with the results described by Arima et al., who reported on the presence of histidine in FF colorectal tissue but not in FFPE samples. Similarly, amino acids involved in tryptophan metabolism were less abundant or absent in FFPE tissue in both their study and ours, while they were readily detectable in FF tissue (Arima et al., 2020). In contrast to our study, they were able to detect isoleucine and leucine. Notably, their method for metabolite extraction differed from ours: they incubated the FFPE samples in methanol at 70 °C, which could have facilitated protein denaturation and, in combination with chemical hydrolysis, promoted the release of free amino acids. This process may have increased the concentration of certain amino acids, making them analytically measurable. In addition, we applied a targeted LC-MS/MS strategy, whereas Arima et al. used an untargeted approach without subsequent targeted validation of the identified metabolites. The absence of validation could potentially have led to inaccuracies in metabolite identification, which may partly explain the differences observed in the amino acids detected in FFPE tissue (Schrimpe-Rutledge et al., 2016).

**Fig. 1:**
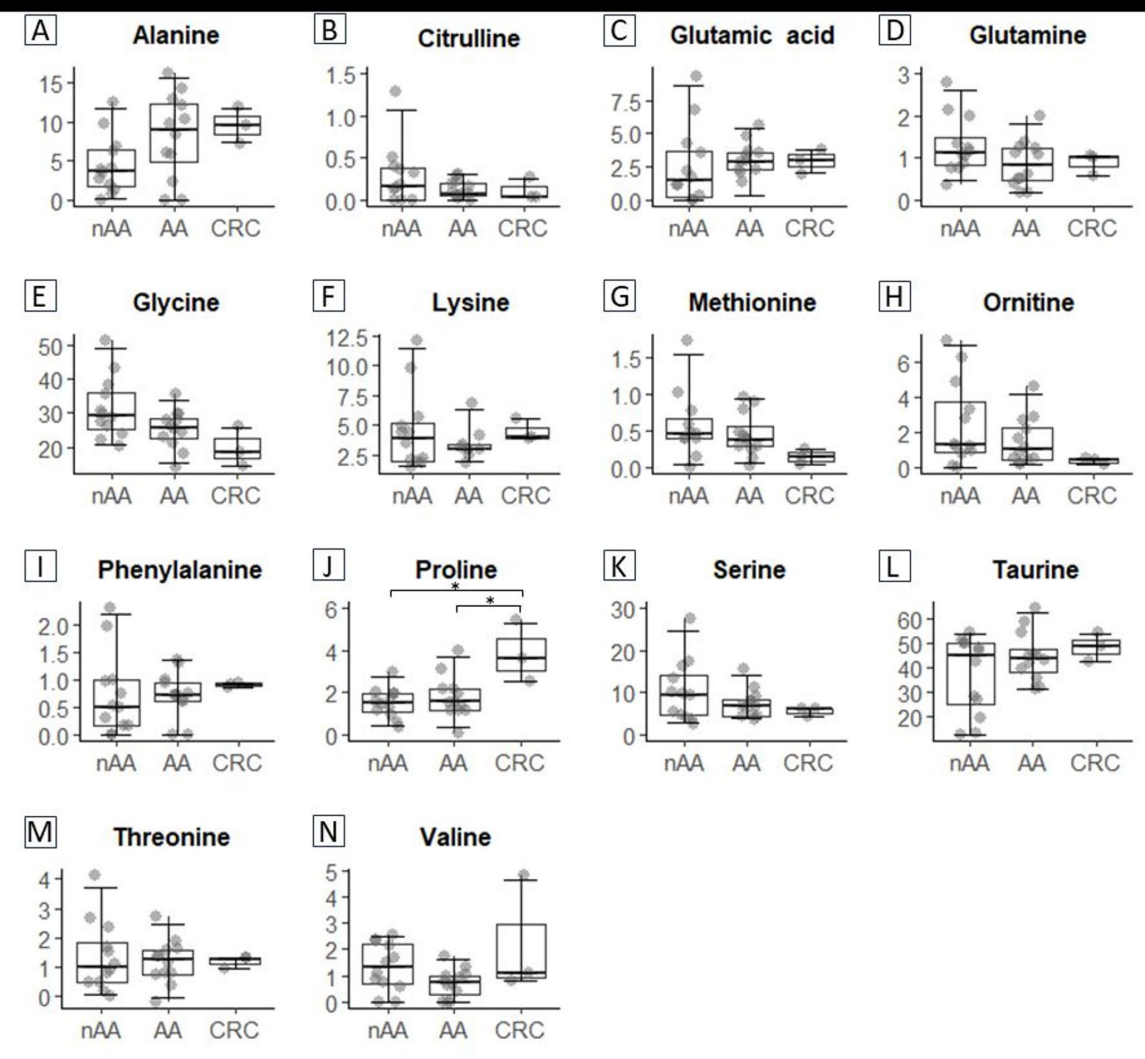
Amino acid concentrations in colorectal neoplasia. Boxplots showing the normalized concentrations of the 14 detected amino acids in nAA, AA and CRC tissue. The y-axis shows the percentage of the targeted amino acid relative to the total amino acids measured. Unpaired univariate analyses were performed using Mann-Whitney U test. Significance levels are depicted with an asterisk: *p < 0.05. *AA* advanced adenoma, *CRC* colorectal carcinoma and *nAA* non-advanced adenoma

To explore multivariable patterns in amino acid concentrations, Principal Component Analysis (PCA) was performed. The first two principal components explained 51.3% of the total variance. A multivariate analysis of variance (MANOVA) on these components showed a significant group effect (Pillai’s trace = 0.555, *F*(4,48) = 2.95, *p* = 0.016), indicating that amino acid profiles differed between diagnostic groups in a multivariable manner. The PCA plot is provided in Supplementary Fig. 1. Univariate analyses showed no significant differences in amino acid concentrations between advanced and non-advanced adenomas (Fig. 1). Glycine and methionine showed decreasing trends along the adenoma-carcinoma sequence, however this was not statistically significant (Fig. 1E, G). A significantly increased relative concentration of proline was detected in CRC compared to AA tissue (FC 2.33, *p* = 0.04) and CRC compared to nAA tissue (FC 2.42, *p* = 0.02) (Fig. 1J). This is in line with many other studies that reported increased concentrations of proline in CRC tissue versus nontumor intestinal tissue and advanced adenoma tissue (Arima et al., 2020; Gao et al., 2016; Mal et al., 2012; Manna et al., 2014). Furthermore, this aligns with our previous findings of an increased proline concentration in the faeces of patients with CRC compared to controls, suggesting its potential as a non-invasive biomarker (Bosch et al., 2022a, b). No associations were observed between storage time and amino acid concentrations, based on Spearman correlation analyses (all *p* > 0.05). In addition, proline concentrations did not differ significantly between male and female patients (*p* = 0.370), despite differences in sex distribution across groups. These findings suggest that neither storage duration nor sex acted as confounding factors in our analyses.

Proline is a key component in cancer biology, playing a central role in various processes considered hallmarks of cancer development (Hanahan, & Weinberg, 2011). Proline biosynthesis, in particular, is essential for tumour growth and energy production. Inhibiting this biosynthetic pathway has been shown to reduce both tumour growth and ATP production (Liu et al., 2015). In addition, proline metabolism is involved in redox regulation, contributing to the formation of reactive oxygen species (ROS), which play key roles in signal transduction for apoptosis and autophagy. At the same time, proline also acts as an antioxidant, protecting cells from ROS-induced damage (Patriarca et al., 2021). Furthermore, proline is essential for extracellular matrix proteins like collagen. The accumulation of interstitial collagens contributes to pathological fibrosis, a feature observed in various tumour tissues (Patriarca et al., 2021). Finally, several studies report a potential role of proline metabolism in cancer initiation and progression. For example, the conversion of pyrroline-5-carboxylate to proline driven by the enzyme pyrroline-5-carboxylate reductase 1 (PYCR1) is reported to be upregulated (Panthong et al., 2024; Westbrook et al., 2022). And studies have demonstrated that proline catabolism via the enzyme proline dehydrogenase (PRODH) is increased in metastases compared to primary tumors in both humans and mice, suggesting that proline catabolism contributes to cancer cell adaptation during the metastatic process (Elia et al., 2017). Inhibiting PRODH may, therefore, represent a potential therapeutic target (Elia et al., 2017).

We acknowledge the limitations of our study. First, the sample size was limited due to the use of a previously consented cohort, which allowed for efficient and ethically approved access to FFPE tissue. Nevertheless, the small sample size resulted in suboptimal statistical power, underscoring the need for external validation of our findings. Second, as snap-frozen tissue was not available, we could not directly assess potential preservation effects; however, all samples underwent identical FFPE processing, allowing for reliable relative comparisons. Furthermore, from a clinical perspective, the addition of healthy intestinal tissue would have enhanced the investigation of amino acids’ role in the adenoma-carcinoma pathophysiology, though it was not available. This is a limitation that will be present when research is conducted on material not specifically collected for scientific purposes. Despite these limitations, the primary aim of this study was to explore the feasibility of measuring amino acids in long-term stored FFPE tissue, a durable and widely available matrix. We successfully detected 14 out of 20 amino acids in small quantities of FFPE tissue that had undergone multiple processing steps and had been stored for an extended period.

## Conclusion

Our findings demonstrate that widely available FFPE colorectal tissue is a suitable matrix for amino acid research, allowing for the detection of 14 out of 20 targeted amino acids, providing a foundation for future studies. Notably, we observed changes in amino acid concentrations along the adenoma-carcinoma sequence, including an increase in proline concentration in colorectal adenocarcinoma compared to advanced- and non-advanced adenoma tissue. Building on this research, future studies may explore a broader range of metabolites or evaluate its applicability across different FFPE tissue types.

## Electronic supplementary material

Below is the link to the electronic supplementary material.

Supplementary Material 1 Supplementary Fig. 1. Principal Component Analysis (PCA) biplot based on normalized amino acid concentrations in FFPE colorectal tissue samples.Points represent individual samples, colored by diagnostic group: non-advanced adenoma (nAA), advanced adenoma (AA), and colorectal carcinoma (CRC). Arrows indicate the loadings of each amino acid on the first two principal components (PC1 and PC2), which together explain 51.3% of the total variance. Ellipses represent the 95% confidence regions for each group. Larger group symbols indicate the group centroids (mean positions of all samples in a group). No ellipse is shown for the CRC group due to the small sample size (n = 3), which prevents reliable estimation. The direction and length of each arrow reflect the contribution of each amino acid to the principal components. Supplementary Table 1. Raw amino acid concentrations per tissue sample mixture. Amino acid concentrations are expressed as µmol/L of tissue sample mixture. Abbreviations: n.d. = not detected

## Data Availability

Data is provided within the supplementary information file.
