# Supplementary material for: Feasibility of amino acid profiling in long-term stored formalin-fixed paraffin-embedded colorectal neoplasia tissue
Source: Metabolomics. 2025 Jul 27;21(4):102. doi: 10.1007/s11306-025-02301-8 (PMC12301268; doi:10.1007/s11306-025-02301-8)
Supplement: Supplementary file 1 — Supplementary Material 1 Supplementary Fig. 1. Principal Component Analysis (PCA) biplot based on normalized amino acid concentrations in FFPE colorectal tissue samples.Points represent individual samples, colored by diagnostic group: non-advanced adenoma (nAA), advanced adenoma (AA), and colorectal carcinoma (CRC). Arrows indicate the loadings of each amino acid on the first two principal components (PC1 and PC2), which together explain 51.3% of the total variance. Ellipses represent the 95% confidence regions for each group. Larger group symbols indicate the group centroids (mean positions of all samples in a group). No ellipse is shown for the CRC group due to the small sample size (n = 3), which prevents reliable estimation. The direction and length of each arrow reflect the contribution of each amino acid to the principal components. Supplementary Table 1. Raw amino acid concentrations per tissue sample mixture. Amino acid concentrations are expressed as µmol/L of tissue sample mixture. Abbreviations: n.d. = not detected [file 11306_2025_2301_MOESM1_ESM.docx]

**Supplementary material**

Feasibility of amino acid profiling in long-term stored formalin-fixed paraffin-embedded colorectal neoplasia tissue

Roza C.M. Opperman^1,2,3^, Puck E. Bruchner^1^, Sofie Bosch^1,2^, Tim G.J. de Meij^2,4,5^, Evelien Dekker^2,3,6^, Nanne K.H. de Boer^1,2^ and Eduard A. Struys^7^

^1^Department of Gastroenterology and Hepatology, Amsterdam UMC, Vrije Universiteit Amsterdam, 1081 HV Amsterdam, The Netherlands.

^2^Amsterdam Gastroenterology Endocrinology Metabolism (AGEM) Research Institute, 1081 HV Amsterdam, The Netherlands.

^3^Cancer Center Amsterdam, research program, 1081 HV Amsterdam, The Netherlands.

^4^Department of Pediatric Gastroenterology, Emma Children's Hospital, Amsterdam UMC, Vrije Universiteit Amsterdam, 1081 HV Amsterdam, The Netherlands.

^5^Department of Pediatric Gastroenterology, Emma Children's Hospital, Amsterdam UMC, Academic Medical Centre, 1105 AZ Amsterdam, The Netherlands.

^6^Department of Gastroenterology and Hepatology, Amsterdam UMC, University of Amsterdam, 1081 HV Amsterdam, the Netherlands.

^7^Department of Laboratory Medicine, Amsterdam University Medical Centre, 1105 AZ Amsterdam, The Netherlands.

**Keywords**: colorectal cancer, colorectal adenoma, amino acids, liquid-chromatography tandem-mass spectrometry, formalin-fixed paraffin embedded

**Correspondence address:** R.C.M. Opperman, MD. Department of Gastroenterology and Hepatology, Amsterdam UMC, Vrije Universiteit Amsterdam, 1081 HV Amsterdam, The Netherlands. ORCiD: 0000-0002-7279-0940 E-mail: [r.opperman@amsterdamumc.nl](mailto:r.opperman@amsterdamumc.nl)

**TABLE OF CONTENTS**

| **Topic** | **Page** |
| --- | --- |
| **Supplementary Figure 1.** Principal Component Analysis (PCA) biplot based on normalized amino acid concentrations in FFPE colorectal tissue samples | 2 |
| **Supplementary Table 1.** Raw amino acid concentrations per tissue sample mixture. | 3 |

**
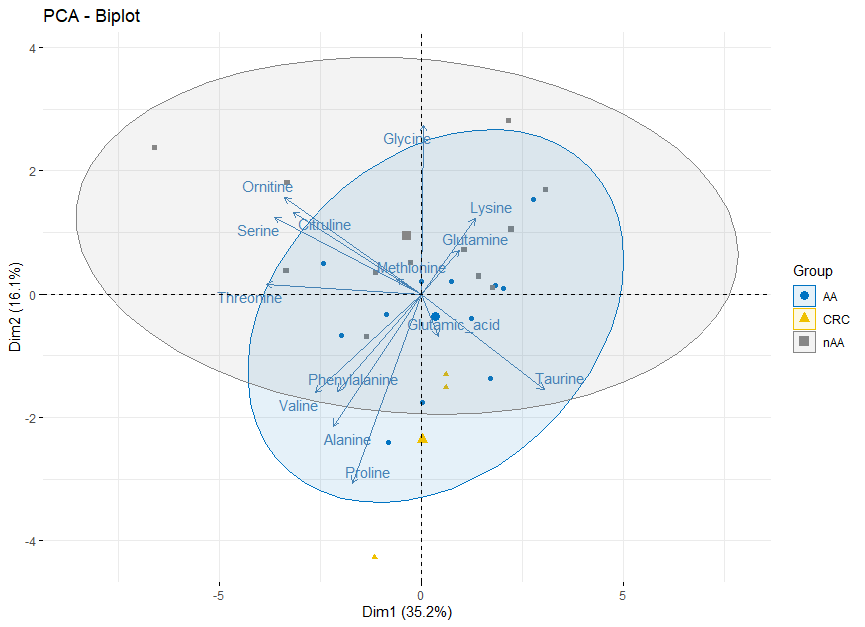
**

**Supplementary Figure 1**. Principal Component Analysis (PCA) biplot based on normalized amino acid concentrations in FFPE colorectal tissue samples.

Points represent individual samples, colored by diagnostic group: non-advanced adenoma (nAA), advanced adenoma (AA), and colorectal carcinoma (CRC). Arrows indicate the loadings of each amino acid on the first two principal components (PC1 and PC2), which together explain 51.3% of the total variance. Ellipses represent the 95% confidence regions for each group. Larger group symbols indicate the group centroids (mean positions of all samples in a group). No ellipse is shown for the CRC group due to the small sample size (*n* = 3), which prevents reliable estimation. The direction and length of each arrow reflect the contribution of each amino acid to the principal components.

**Supplemental table 1.** Raw amino acid concentrations per tissue sample. ^a^

| Sample | Glycine | Threonine | Alanine | Glutamic acid | Proline | Taurine | Glutamine | Citrulline | Serine | Phenylalanine | Valine | Methionine | Ornithine | Lysine |
| --- | --- | --- | --- | --- | --- | --- | --- | --- | --- | --- | --- | --- | --- | --- |
| CRC_1 | 3.478 | 0.242 | 1.347 | 0.556 | 0.468 | 10.149 | 0.199 | 0.050 | 1.113 | 0.173 | 0.150 | 0.007 | 0.077 | 0.722 |
| CRC_2 | 5.410 | 0.506 | 4.453 | 0.739 | 2.044 | 18.371 | 0.216 | 0.015 | 2.257 | 0.352 | 1.808 | 0.054 | 0.065 | 1.499 |
| CRC_3 | 4.856 | 0.173 | 1.780 | 0.707 | 0.677 | 7.850 | 0.186 | 0.005 | 0.790 | 0.159 | 0.200 | 0.047 | 0.098 | 1.042 |
| AA_1 | 3.400 | 0.189 | 1.399 | 0.895 | 0.390 | 15.570 | 0.145 | 0.016 | 1.029 | 0.148 | 0.092 | 0.060 | 0.218 | 0.719 |
| AA_2 | 2.995 | 0.167 | 1.299 | 0.244 | 0.231 | 3.844 | 0.133 | 0.017 | 0.859 | 0.107 | 0.098 | 0.101 | 0.289 | 0.323 |
| AA_3 | 4.767 | 0.320 | 3.148 | 0.551 | 0.774 | 7.651 | 0.245 | 0.009 | 1.366 | 0.252 | 0.260 | 0.155 | 0.034 | n.d. |
| AA_4 | 2.272 | 0.143 | 1.076 | 0.173 | 0.167 | 2.400 | 0.037 | 0.016 | 0.849 | 0.104 | 0.131 | 0.036 | 0.219 | n.d. |
| AA_5 | 3.145 | 0.091 | 0.932 | 0.544 | 0.217 | 4.837 | 0.223 | 0.010 | 0.508 | 0.084 | 0.074 | 0.033 | 0.031 | 0.468 |
| AA_6 | 2.241 | 0.115 | 0.513 | 0.282 | 0.101 | 3.894 | 0.093 | 0.026 | 0.686 | 0.062 | 0.088 | 0.031 | 0.183 | 0.210 |
| AA_7 | 3.411 | 0.192 | 2.084 | 0.477 | 0.504 | 7.282 | 0.183 | 0.010 | 1.441 | 0.118 | 0.128 | 0.048 | 0.082 | 0.297 |
| AA_8 | 0.485 | 0.019 | 0.060 | 0.151 | 0.030 | 1.203 | 0.014 | 0.000 | 0.100 | 0.000 | 0.000 | 0.012 | 0.030 | 0.570 |
| AA_9 | 0.339 | 0.000 | 0.000 | 0.000 | 0.000 | 0.518 | 0.002 | 0.000 | 0.060 | 0.000 | 0.000 | 0.000 | 0.004 | 0.032 |
| AA_10 | 0.737 | 0.013 | 0.000 | 0.043 | 0.045 | 1.906 | 0.005 | 0.002 | 0.138 | 0.023 | 0.021 | 0.028 | 0.054 | 0.223 |
| AA_11 | 2.063 | 0.220 | 0.837 | 0.171 | 0.124 | 2.591 | 0.032 | 0.024 | 1.255 | 0.075 | 0.079 | 0.031 | 0.379 | 0.249 |
| AA_12 | 0.888 | 0.041 | 0.292 | 0.107 | 0.036 | 1.268 | 0.041 | 0.006 | 0.204 | 0.018 | 0.000 | 0.004 | 0.014 | 0.091 |
| nAA_1 | 0.331 | 0.003 | 0.040 | 0.000 | 0.002 | 0.174 | 0.007 | 0.000 | 0.024 | 0.001 | 0.000 | 0.000 | 0.000 | 0.063 |
| nAA_2 | 0.527 | 0.002 | 0.018 | 0.093 | 0.008 | 0.590 | 0.038 | 0.000 | 0.034 | 0.000 | 0.000 | 0.008 | 0.000 | 0.061 |
| nAA_3 | 0.857 | 0.077 | 0.358 | 0.269 | 0.057 | 0.381 | 0.031 | 0.010 | 0.475 | 0.028 | 0.067 | 0.011 | 0.142 | 0.125 |
| nAA_4 | 0.587 | 0.039 | 0.056 | 0.005 | 0.049 | 0.323 | 0.033 | 0.008 | 0.286 | 0.017 | 0.042 | 0.006 | 0.105 | 0.093 |
| nAA_5 | 0.651 | 0.022 | 0.021 | 0.058 | 0.039 | 1.365 | 0.033 | 0.000 | 0.129 | 0.004 | 0.020 | 0.021 | 0.036 | 0.329 |
| nAA_6 | 1.690 | 0.243 | 0.568 | 0.065 | 0.121 | 0.762 | 0.021 | 0.076 | 1.627 | 0.030 | 0.127 | 0.027 | 0.427 | 0.115 |
| nAA_7 | 0.262 | 0.007 | 0.024 | 0.000 | 0.010 | 0.172 | 0.005 | 0.000 | 0.057 | 0.014 | 0.014 | 0.010 | 0.007 | 0.021 |
| nAA_8 | 0.958 | 0.056 | 0.069 | 0.064 | 0.048 | 1.790 | 0.027 | 0.005 | 0.380 | 0.027 | 0.041 | 0.038 | 0.122 | 0.069 |
| nAA_9 | 4.014 | 0.117 | 0.532 | 0.461 | 0.154 | 6.590 | 0.153 | 0.016 | 0.692 | 0.039 | 0.073 | 0.018 | 0.121 | 0.194 |
| nAA_10 | 1.655 | 0.028 | 0.165 | 0.256 | 0.057 | 3.292 | 0.080 | 0.011 | 0.210 | 0.029 | 0.051 | 0.028 | 0.046 | 0.123 |
| nAA_11 | 0.089 | 0.000 | 0.000 | 0.000 | 0.008 | 0.223 | 0.009 | 0.001 | 0.058 | 0.000 | 0.007 | 0.013 | 0.005 | 0.022 |
| nAA_12 | 0.707 | 0.054 | 0.221 | 0.038 | 0.051 | 1.526 | 0.028 | 0.013 | 0.312 | 0.063 | 0.048 | 0.013 | 0.092 | 0.071 |

^a^ Amino acid concentrations are expressed as µmol/L of tissue sample mixture. Abbreviations: n.d. = not detected.
